# Nocturnal digital surveillance in aged populations and its effects on health, welfare and social care provision: a systematic review

**DOI:** 10.1186/s12913-021-06624-9

**Published:** 2021-06-30

**Authors:** Matt X. Richardson, Maria Ehn, Sara Landerdahl Stridsberg, Ken Redekop, Sarah Wamala-Andersson

**Affiliations:** 1 School of Health and Welfare, Mälardalen University, Box 325, 631 05 Eskilstuna, Sweden; 2 School of Innovation, Design and Engineering, Mälardalen University, Västerås, Sweden; 3 School of Health and Welfare and the Institutional Library, Mälardalen University, Västerås, Sweden; 4 Erasmus University, Rotterdam, the Netherlands

**Keywords:** Health and welfare technology, Nocturnal surveillance, Remote monitoring, Aging, Elderly

## Abstract

**Background:**

Nocturnal digital surveillance technologies are being widely implemented as interventions for remotely monitoring elderly populations, and often replace person-based surveillance. Such interventions are often placed in care institutions or in the home, and monitored by qualified personnel or relatives, enabling more rapid and/or frequent assessment of the individual’s need for assistance than through on-location visits. This systematic review summarized the effects of these surveillance technologies on health, welfare and social care provision outcomes in populations ≥ 50 years, compared to standard care.

**Method:**

Primary studies published 2005–2020 that assessed these technologies were identified in 11 databases of peer-reviewed literature and numerous grey literature sources. Initial screening, full-text screening, and citation searching steps yielded the studies included in the review. The Risk of Bias and ROBINS-I tools were used for quality assessment of the included studies.

**Result:**

Five studies out of 744 identified records met inclusion criteria. Health-related outcomes (e.g. accidents, 2 studies) and social care outcomes (e.g. staff burden, 4 studies) did not differ between interventions and standard care. Quality of life and affect showed improvement (1 study each), as did economic outcomes (1 study). The quality of studies was low however, with all studies possessing a high to critical risk of bias.

**Conclusions:**

We found little evidence for the benefit of nocturnal digital surveillance interventions as compared to standard care in several key outcomes. Higher quality intervention studies should be prioritized in future research to provide more reliable evidence.

**Supplementary Information:**

The online version contains supplementary material available at 10.1186/s12913-021-06624-9.

## Background

Nocturnal digital surveillance systems are health and welfare technology interventions used to monitor aged populations, both with and without cognitive or physiological dysfunctions, in the home or institutional care settings. Such systems generally use sensors or cameras to determine if an individual is present at a specific location in the night, and upon deviation from a normal, “desired” status sends a message or alarm to an external, specified resource such as a formal or informal caregiver. In other cases, the surveillance system replaces an external resource that would have physically visited the individual during the night, often on a pre-determined schedule, to ensure they did not require assistance; the individual is instead observed digitally. The external resource following-up on or receiving messages regarding the individual can then assess if any action is required to ensure their safety and well-being.

The conceivable advantages to such surveillance interventions are several. The disturbance created by physically visiting individuals during their sleep is conceivably reduced since a digital monitor can be remotely and silently activated. The constancy of the surveillance system is another, as adverse events may occur between intermittent physical visits and not be discovered until a significant period has elapsed. Users might feel an increased sense of security or reduced level of worry, allowing sleep quality to improve. Public services may expect downstream cost-savings due to reductions in resource-intensive operations, such as reducing the need for employees to visit individuals throughout the night. The costs for personnel, transport, and administration would conceivably decrease if distance-based surveillance could perform the same tasks. Furthermore, during the Covid-19 pandemic, the interest in and use of these systems has increased substantially to reduce physical contact with populations at greater risk of serious illness, while still maintaining a necessary standard of care.

Developments in Sweden provide an indication of the increasing usage of digital night surveillance. The number of municipalities that provide such technologies in individual residences has increased from 23 to 2016 to 66 in 2020, and for institutional residences from six to 44 over the same period. Of Sweden’s 218 municipal elderly care services, 66 % are currently using digital surveillance interventions in the home, as are 33 % of public services responsible for care of the functionally disabled [1]. Institutional use is even more prevalent; 90 % of elderly care institutions in Sweden use some form of digital nocturnal surveillance intervention, as do 80 % of other types of group residences [1]. The estimated potential economic savings for nationally implementing digital night surveillance in elderly care is 1,4 billion SEK, or 140 SEK per individual [2]. The municipality of Gothenburg estimated that annual savings amounted to 71,000 SEK per user after implementing such a system for elderly persons living at home, compared to nightly visits [3].

Considering the increasing use of these interventions, the importance of assessing outcomes of interest is also increasing in order to determine the actual and potential benefits to individuals, their informal caregivers, and public services.

### Objective

This systematic review assessed the effects of nocturnal digital surveillance on the health, welfare and social care provision in aged populations compared to standard care.

## Methods

### Protocol and registration

This systematic review was prospectively registered in the PROSPERO international prospective register of systematic reviews (reg CRD42020198438, September 11, 2020). This register (https://www.crd.york.ac.uk/prospero/) was searched for ongoing or previously published reviews with similar topics and criteria before registering our own review and initiating the search strategy; none were identified as overlapping.

### Inclusion criteria

The specified population for the review was adults 50 years of age and older residing in the home or non-penal care institutions, in Organisation for Economic Co-operation and Development (OECD) countries or those of an equivalent level of development. Interventions that could be included were digital cameras, sensors, alarms, or other place-based, non-physiological monitoring devices that were used specifically during the night. The intervention(s) were compared with standard care, typically no surveillance or on-site, person-based surveillance. Outcomes of interest were:

- Health-related outcomes, including rate of injury, unexplained absence, and other adverse medical outcomes.
- Welfare outcomes, including quality of life, perceived safety or security, and other related welfare outcomes.
- Social care provision outcomes including the welfare of, and burden on, families, informal caregivers and social care staff and organisations, as well as related economic outcomes for public services (e.g. transportation, search costs).

The included study types were randomized control trials (RCTs) including randomized cross-over trials, cluster randomized trials, and within-subjects designs, as well as quasi-experimental studies including non-randomized control studies, before-and-after studies, and interrupted time series. Mixed-methods studies that included relevant quantitative data were included. Grey literature including reports from public agencies, theses, and conference abstracts with similar study designs as for the peer-reviewed literature were also included. Studies must have been published between January 2005 (when the digital nocturnal surveillance technologies in focus generally became established) and August 2020, in English, French, or Scandinavian languages.

### Exclusion criteria

Proof-of-concept, conceptual, qualitative studies (that e.g. described attitudes or feasibility without providing any relevant quantitative outcome data) and observational studies that did not adjust or *a priori* identify any independent variable were excluded from the review. Systematic, literature and scoping reviews, as well as other grey literature not reporting primary study results were excluded.

Hospitalized populations were excluded. Interventions intended for continuous monitoring of specific physiological conditions (i.e. apnea, seizure, heart rate etc.) were not included, nor were interventions outside the home or institutional setting or used for daytime surveillance. In the case of combined daytime/nighttime surveillance interventions, if the data for outcomes could not be extracted solely for nocturnal settings then the study was excluded.

### Search strategy

#### Peer-reviewed literature

The following databases were initially searched between July and August 2020 for peer-reviewed literature: PubMed (pubmed.ncbi.nlm.nih.gov), Cinahl Plus (EBSCOhost), Cochrane Library (www.cochranelibrary.com), Web of Science (www.webofknowledge.com), IEEE Xplore (ieeexplore.ieee.org), APA Psycinfo (EBSCOhost), Academic Search Elite (EBSCOhost), Applied Social Sciences Index and Abstracts-ASSIA (ProQuest), International Bibliography of the Social Sciences – IBSS (ProQuest), Scopus (www.scopus.com), and SocIndex (EBSCOhost). Citation searches following initial searches were conducted in August and September 2020 (see Study Selection Process for further explanation).

#### Grey literature

Searches in the grey literature were conducted in September 2020 in the following international registers and databases of grey literature: OpenGrey (www.opengrey.eu), OAlster (oaister.worldcat.org), Bielefeld Academic Search Engine – BASE (www.base-search.net), WHO ICTRP (apps.who.int/trialsearch), ClinicalTrials.gov (clinicaltrials.gov/ct2/home), the International Health Technology Assessment Database (https://www.inahta.org/hta-database), DART-Europe (www.dart-europe.eu/basic-search.php), and Dissertations and Theses A&I (ProQuest). National sources of grey literature in the Nordic countries including publications and websites of public agencies, national databases, NGOs and interest groups were also searched in October 2020 using search strings in the respective Scandinavian languages. A complete list of the grey literature sources searched with links to websites is included in Supplement 1.

Searches in both types of information sources were followed up with searches in Google Scholar in English, Swedish, Norwegian, Danish, and Finnish languages.

### Search strings

#### Peer-reviewed literature

The following search string was initially used in PubMed, and then translated in the searches of the other peer-reviewed literature databases:

(elderly OR “older adult*” OR “older person*” OR aged (MeSH only databases)) AND (nocturnal OR “night-time” OR “nighttime” OR “night time”) AND (surveillance OR camera* OR “video monitor*” OR “in-home monitor*” OR “home monitor*” OR “safety monitor*” OR “digital monitor*” OR telemonitor* OR “remote monitor*” OR “digital camera” OR “digital sensor*” OR “monitoring system*”).

The search string was adjusted in those databases that imposed limitations on search fields, operators and/or wildcards, so that several overlapping searches may have been required.

#### Grey literature

If the database allowed, the search string was identical to that used in the peer-reviewed literature searches. However, many databases did not allow advanced search strings, in particular websites of e.g. public agencies. The search string from the peer-reviewed literature was therefore divided into its components (delineated by the AND operator in that search string), or if necessary, into individual keywords. Screening of clearly irrelevant literature in these cases was conducted during the search.

An example of this was a grey literature database with a search maximum of 5 terms and without phrase searching. The resulting records identified at each step in the search strategy were then opened, and the search terms re-combined in-document to determine inclusion in the study selection process:

Grey literature database search (maximum 5 terms), with results downloaded after each step.

- Search 1: elderly OR older adult OR older person OR aged.
- Search 2: nocturnal OR night-time OR nighttime OR night.
- Search 3: surveillance OR camera OR video monitor OR in-home monitor OR home monitor.
- Search 4: safety monitor OR digital monitor OR telemonitor OR remote monitor OR digital camera.
- Search 5: digital sensor OR monitoring system.

Presence of any of terms in 1 AND any terms in 2 AND any terms in (3 or 4 or 5) in a publication: included in study selection process.

### Study selection process

**Fig. 1:**
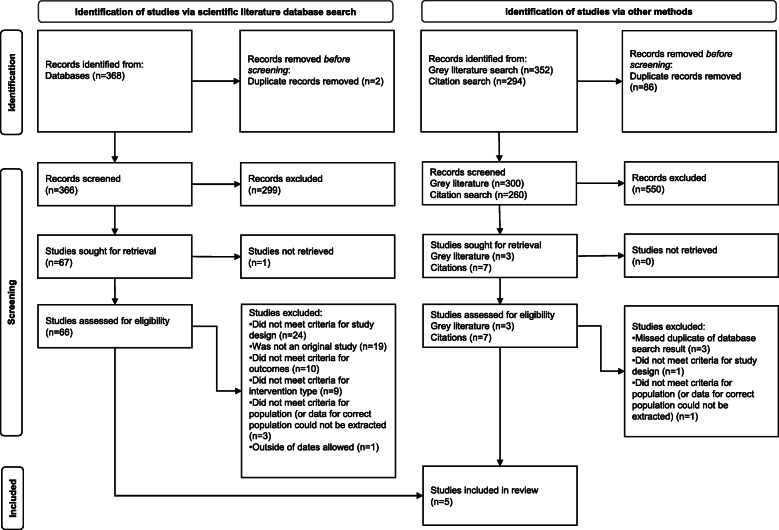
PRISMA flowchart for selection of studies.

All identified records were entered into the Covidence systematic review software (Veritas Health Innovation Ltd, Melbourne, Australia, www.covidence.org) during the review process. The PRISMA guidelines for reporting of systematic reviews [4] were followed and a summary of the study selection results can be found in Fig. 1.

Two researchers (MXR and ME) and one information sciences specialist (SLS), hereafter referred to as reviewers, conducted the review process which had four steps:

#### Initial screening

The titles, keywords, and in some cases abstracts of the obtained records were screened for relevance by two reviewers independently. Each reviewer voted on whether the record was relevant for further review; consensus resulted in inclusion or exclusion at this step. Any remaining conflicts were resolved by the third reviewer.

#### Full-text screening

The full text for all studies proceeding to this step were obtained and read independently by two reviewers. Each reviewer voted on whether the study was relevant for inclusion in the review and to proceed to data extraction. Consensus resulted in inclusion or exclusion at this step, and any conflicts remaining were resolved by the third reviewer.

#### Citation searching

We searched the cited references of review-type publications that proceeded past the screening stage but were then excluded as they were not original studies. We also searched Scopus for articles citing the same review-type publications. The cited references for, and citations of, any studies included after the full-text screening step were also searched in the same manner. Both the cited and the citing articles followed the steps preceding this one in the review process.

#### Data extraction

Essential information regarding the study aim, design, conduct, population, intervention, and outcomes, as well as results data for relevant outcomes, was extracted from the study by two reviewers independently. The template for this extraction can be found in Supplement 2. Consensus resulted in inclusion of the extracted data in the review’s summary of findings. Conflicts in extraction were discussed among the two reviewers, and any remaining conflicts remaining were resolved by the third reviewer.

#### Risk of bias assessment

The included individual studies were assessed for risk of bias at the study level by two researchers independently. The criteria assessed for randomized studies followed the Risk-of-bias tool [5] and included the method for random sequence generation, allocation concealment, blinding of participants and personnel, blinding of outcome assessment, the completeness of outcome data, the possibility of selective outcome reporting, and other sources of bias. For non-randomized studies, the ROBINS-I tool [6] was used to assess bias due to confounding, missing data and selective reporting, and in selection of participants, classification of and deviations from interventions. Consensus about the risk of bias resulted in inclusion of the risk assessment in the review’s summary of findings. Conflicts in risk assessment were discussed among the two reviewers, and any conflicts remaining were resolved by the third reviewer.

### Reported measures

The main statistics in each study were reported. Further synthesis of results from the studies was not possible due to lack of statistical reporting in the two studies found in the grey literature, where p-values and descriptive statistics were not reported, and the diversity of outcomes.

## Results

### Study selection

Altogether, 926 records (366 from initial scientific literature database searches, 260 from subsequent citation searches, and 300 from grey literature searches) were identified after removal of duplicates (Fig. 1).

### Study characteristics

Of the five included studies, three were peer-reviewed (Holmes et al., 2007[7]; Rowe et al. 2009 [8] and 2010 [9]); of these, two were randomised controlled trials [8, 9], and one was a variant of a cluster randomized trial (also described by the study’s authors as “quasi-experimental”) [7]. The final two studies (Sivertsen and Løe, 2019 [10]; Røhne et al., 2016 [11]) were found in the grey literature and had non-randomized, mixed methods designs. A summary of the study characteristics is presented in Table 1.

**Table 1:** Summary of characteristics for studies included in the review.^a^

Country	Author, year, and title	Intervention	Participants^b^	Design	Duration of intervention	Comparator
USA	Holmes, D et al., 2007, “An evaluation of a monitoring system intervention: falls, injuries and affect in nursing homes” [7]	Surveillance package including bed exit sensor and bathroom/bedroom exit monitors	92 primarily female, white and widowed care institution residents, age 87 (7.5)	Variant of cluster randomized trial	15 months	On-location visits
USA	Rowe, M et al., 2010, USA, “Sleep in dementia caregivers and the effects of a nighttime monitoring system” [8]	Surveillance package including bed occupancy sensor and room movement detector, emergency door alarm	53 persons with dementia (age 79 (8.4), 50 % female) and their informal caregivers (age 62 (11.9), 81 % female, 70 % white and 18 % black; 51 % spouses and 38 % adult daughters).	Randomized controlled trial	12 months	No nighttime monitoring system
USA	Rowe, M et al., 2009, “Reducing dangerous nighttime events in persons with dementia using a nighttime monitoring system” [9]	Surveillance package including bed occupancy sensor and room movement detector, emergency door alarm	Same as Rowe et al., 2010	Randomized controlled trial	12 months	No nighttime monitoring system
Norway	Sivertsen and Løe, 2019, “Digitalt nattilsyn og søvnkartlegging: evaluering av digital nattilsyn på REKO Kastvollen” (*Digital night surveillance and sleep mapping: evaluation of digital night surveillance at REKO Kastvollen*) [10]	Bed supervision system	20 care institution employees; detailed demographic information not stated	Mixed methods, within-subjects design	9 months	On-location visits
Norway	Røhne et al., 2016, “Effekt av Trygghetspakker: erfaringar fra velferdsteknologiprosjektet i Lister regionen” (*Effect of security packages: experiences from the welfare technology project in Lister region*) [11]	Surveillance package including bed, safety and door alarms	9 elderly persons living at home and their caregiving public employees; detailed demographic information not stated	Mixed methods, within-subjects design	9 months	On-location visits

^a^Holmes et al. (2007) was found in PubMed Academic Search Elite, Cinahl, Cochrane (Trials), ASSIA, and Scopus; Rowe et al. (2010) in PubMed, Academic Search Elite, Cinahl, APA PsycInfo, Cochrane (Trials), Web of Science, ASSIA, and Scopus; Rowe et al. (2009) in PubMed, Academic Search Elite, Cochrane (Trials), Web of Science, ASSIA, and Scopus; and Siwertsen and Løe (2019) and Røhne et al. (2016) in Nordic grey literature databases

^b^Number of participants is presented; mean age (standard deviation in parentheses) and/or additional description presented where available

### Risk of bias assessment within studies

Summaries of the assessed risk of bias for individual randomised and non-randomised studies are presented in Tables 2 and 3, respectively.

**Table 2:** Risk of bias assessments for individual randomized studies according to standardized criteria (Risk of Bias tool [5, 12]). A minus sign (-) indicates a high risk of bias assessment, and a plus sign (+) indicates a low risk of bias assessment for the randomized studies. A question mark (?) indicates some concerns of bias due to insufficient documentation or information. No symbol indicates the type of bias could not be assessed

Randomized studies (RoB tool assessment criteria)	Holmes et al., 2007 [7]	Rowe et al., 2010 [8]	Rowe et al., 2009 [9]
Random sequence generation	-	-^b^	-^c^
Allocation concealment	-	-	-
Blinding of participants / personnel	-	-	-
Blinding of outcome assessment	-	-	-
Incomplete outcome data	-	-	+
Selective reporting	+	+	+
Other bias	?^a^		
Comments	^a^ staff showed “resistance” to the intervention and altering methods of care	^b^ 8 participants were exempted from randomization according to preference or previous study participation	^c^ 8 participants were exempted from randomization according to preference or previous study participation

**Table 3:** Overall risk of bias assessments for individual non-randomized studies according to standardized criteria (ROBINS-I [6]), using the tools’ ascending scale of risk (low, moderate, serious, critical, or No Information (NI), meaning the risk of bias could not be assessed)

Non-randomized studies (ROBINS-I assessment criteria)	Sivertsen and Løe, 2019 [10]	Røhne et al., 2016 [11]
Bias due to confounding	Critical	Serious
Bias in selection of participants	NI	NI
Bias in classification of interventions	Moderate	Serious
Bias in deviations from intervention	Low	Low
Bias due to missing data	NI	Critical
Bias in measurement of outcomes	Serious	Serious
Bias in selective reporting	Moderate	Moderate
Overall Bias	Critical	Critical
Comments/direction of bias	Unpredictable	Unpredictable

### Summary of findings

The summary of findings for outcomes of the included studies is presented in Table 4. The two studies assessing health-related outcomes did not find any difference in rate of injury or dangerous events when using nocturnal digital surveillance, compared to no surveillance or on-location, person-based surveillance. Two studies found that welfare outcomes improved for either persons under surveillance or their relatives. Four studies assessed social care provision outcomes such as caregiver burden; of these, one study found that work effectiveness and processes improved among 75 % of employees using nocturnal digital surveillance compared to person-based surveillance. One study found that costs for social care provision by a caregiving organisation were reduced with nocturnal digital surveillance compared to on-location, person-based surveillance. The statistics provided in Table 4 are the same as those presented in the studies.

**Table 4:** Summary of findings for outcomes of interest in the systematic review, with overall risk of bias assessment

Nocturnal digital surveillance				
**Population**: Persons ≥ 50 years of age **Settings**: living at home or in care settings in OECD countries or equivalent **Intervention**: digital sensors or monitors used at night for non-physiological surveillance **Comparison**: Care as usual				
**Outcomes**	**Number of studies**	**Relative change due to intervention**	**Overall risk of bias**^**a**^	**Comments**
**Health-related outcomes**: injury, unexplained absence, other adverse medical outcome for receivers of care	2	1: No difference in rates of injury (0.02, *p* = 0.828; Holmes et al. 2007) [7] 2: No difference in reduction of dangerous events (X^2^ = 1.72; *p* = 0.79 for whole period; X^2^ = 3.58; *p* = 0.058 for when system turned on; Rowe et al. 2009) [8]	High	192 accidents and 9 dangerous events, respectively, were observed during the two studies.
**Welfare outcomes**: quality of life, perceived safety or security, and other related welfare outcomes for receivers of care	2	1: Improved affect (mean change in 5-point affect scale: 0.29, *p* = 0.034; Holmes et al. 2007) [7] 2: 95 % reported some improvement in quality of life (Sivertsen and Løe, 2019) [10]	High	Improved affect (in Holmes et al. 2007) [7] was directly predicted by increased direct care due to increased surveillance. Perceived QoL was reported by caregivers for patients (in Sivertsen and Løe, 2019) [10] based on a single survey question.
**Social care provision outcomes**: burden informal caregivers and social care staff and organisations	4	1,2: No evidence of a difference in staff burden (-0.02, *p* = 0.96; Holmes et al. 2007; no statistic for Røhne et al. 2016) [11] 3: No evidence of a difference in caregiver worry (0.72; *p*-value not reported), sleep time (-3.91, *p* = 0.20) or quality (0.05, p-value not reported; all from Rowe *et al. 2010*) [8] 4: 75 % reported improvement in work effectiveness and processes (Sivertsen and Løe, 2019) [10]	High	Staff burden (in Holmes et al. 2007) [7] was a monthly average of daily scores on a seven-item, 3- or 5-point Likert scale. Caregiver worry measured on a 10-point Likert scale; objective sleep by actigraphy; sleep quality on a 5-point Likert scale (all from Rowe et *al*, 2010) [8] Improvement in work effectiveness and processes was reported (in Sivertsen and Løe, 2019) [10] based on a single survey question.
**Social care economic outcomes**: e.g. costs for visiting and transportation, search costs when missing.	1	15 % reduction in social care costs (Røhne et al. 2016) [11]	High	Cost reductions were associated with fewer required visits and transportation; avoided costs due to ability to remain in the home instead of care institutions were not assessed.
^**a**^**High** = There is a high risk that bias was introduced during the study that affects the certainty of the outcome and lowers confidence in the result. This assessment is equivalent to “High” for the Risk of Bias tool, or Serious/Critical in the ROBINS-I tool. **Low** = There is a low risk that bias introduced during the study will affects the certainty of the outcome and lowers confidence in the result. This assessment is equivalent to “Low” for the Risk of Bias tool, or Low/Moderate in the ROBINS-I tool.				

### Risk of bias across studies

Publication bias or other types of bias that may have affected the cumulative evidence were not systematically assessed in this review due to the low number of identified studies in the peer-reviewed literature.

## Discussion

### Summary of evidence

This review aimed to determine if nocturnal digital surveillance affects health-related, welfare and social care provision outcomes in aged populations compared to standard care. The included studies suggest there is currently little evidence that a reduction in adverse health-related outcomes of care receivers can be achieved by using digital surveillance at night. Furthermore, the burden on informal caregivers and formal care staff does not appear to be reduced by using digital night surveillance, and only one study reported that work processes may be more effective in some cases. Evidence for improvement in quality of life for care receivers was also found in only one study regarding affect, and in a second regarding quality of life overall, although the latter was associated with a more direct level of care associated with increased surveillance. Cost reductions for formal social care services also appears possible, by reducing the number of required visits and transportation at night.

The quality of all included studies was low, however, which greatly affects the certainty of the outcomes and results. Several studies included in the current review failed to measure or assess potentially confounding or modifying factors such as age distribution, presence of illness or dementia in non-homogenous groups, the pre-assessed need for institutional care, availability of informal caregivers, and the geographic location of the user in relation to formal and informal care providers. Statistical reporting in the grey literature studies was not at the standard required of peer-reviewed studies, and they did not use standardized or validated measurement tools in some cases.

The lack of high-quality peer-reviewed literature that specifically assessed concrete outcomes of digital night surveillance methods is surprising, considering the widespread use of and reliance upon these interventions. The methods required to evaluate the effects of such interventions are straightforward and can follow established study designs with low risk of bias without difficulty, which makes their absence in the literature perplexing. Indeed, an earlier literature review of all types of monitoring technologies and their outcomes in independently living elderly people [13] supports this conclusion. In that review, the authors stated that research was widespread, but out of 141 included studies only four provided longitudinal data and only one was a RCT. Most research focused on the accuracy, sensitivity and specificity of monitoring systems, failing to evaluate health-related effects of the end-users. The grey literature also yielded a substantial number of intervention assessments, however most lacked formal study designs or approaches that involved manipulation or comparison of variables, excluding them from final compilation despite the current review’s broader inclusion criteria.

There are several possible explanations for the lack of high-quality literature assessing digital nocturnal surveillance. During the literature searches, several studies and reports could not be included in the review as they assessed multiple interventions, typically in the form of “safety packages”, in a manner that did not allow assessment or reporting of the effects of individual components. Some of these studies included a form of nocturnal monitoring, but any effects may have been due to other daytime monitoring systems or active assistance interventions. Without the ability to identify which components have a positive effect on individuals receiving care, there is potential loss of health benefits and an increased risk of unnecessary expenses for care providers and public services.

Proof-of-concept studies of prospective nocturnal monitoring systems were abundant in the literature searches, suggesting that there is a considerable interest in further introducing similar interventions to the health and welfare sector. There was also a plethora of qualitative studies assessing the acceptance and feasibility of market-ready digital monitoring systems, suggesting that there was imminent intention to introduce such interventions. Finally, there were several studies specifically assessing the implementation processes involving monitoring technologies, suggesting that many interventions were indeed being used in society. That the number of these studies appeared to greatly outweigh studies of effects of the interventions in question is a fascinating result of this current review.

The lack of effect evaluation research of nocturnal digital monitoring might be explained if such interventions are simply viewed as a lower-cost alternative to current monitoring methods, namely physical visits by employees and the associated time and or transportation costs. While one article in the current review states that this is (likely) the case, adverse events were not reported as an outcome – and these are likely the costliest aspects associated with, and hopefully prevented by, any kind of monitoring. That article [11] and three others [7, 8, 10] in the review, did not find that employee burden decreased, either. While costs of initial purchase, implementation, training and establishment of centralized functions were not explicitly assessed in any of the articles, if a digital monitoring system does not result in a more favorable ratio between monitoring accuracy and work burden, then the prospective cost savings of a less person-intensive system run the risk of being negated by the increased need to deal with adverse events. Without established evidence to support implementation, care-providing organizations may, in contrast to their intentions, fail to achieve greater work efficiency and economic gain.

Health and welfare technologies are *“technology-based interventions that aim at maintaining or promoting health, wellbeing, quality of life and/or increasing efficiency in the service delivery system of welfare, social and health care services, while improving working conditions of the staff” *[14]. While these types of interventions are not new, current evidence assessment frameworks for other types of clinical interventions are often not applied, or in other cases not fit-for-purpose to a degree that allows meaningful assessment. The UK-based National Institute for Health and Care Excellence (NICE) has however developed one such framework for Digital Health Technologies, in which interventions are classified by their intended functions and then associated with “tiers” of required evidence [15]. The authors of this review followed the guidelines provided by NICE in order to categorize the nocturnal digital surveillance interventions in this review, and judged them as tier 3b, “active monitoring”. Such technologies “automatically record information and transmit the data to a professional, caregiver or third-party organization, without any input from the user, to inform clinical management decisions”, and examples such as sensors in the home are given. For this tier, NICE recommends high-quality intervention studies as a minimum evidence standard, and high-quality randomized controlled studies with validated condition-specific outcome measures in a locally relevant setting as a best practice evidence standard. The result of this current review shows that this higher standard of evidence for nocturnal digital surveillance interventions is not currently obtainable from the broader literature.

### Implications for policy and practice

The results of this review suggest the need for greater explicitness in identifying outcomes of greatest importance to stakeholders prior to implementing a health and welfare technology intervention. This would facilitate assessment of both intended outcomes and unintended consequences and clarify evidence requirements in decision-making processes. While these interventions may be more heavily relied upon during the COVID-19 pandemic in order to reduce the risk of disease transmission, their effectiveness likely needs to be re-assessed when “normal” usage situations are once again established.

### Limitations

This systematic review excluded daytime digital monitoring interventions. Several studies excluded from review may have assessed interventions that could be applicable both day and night, as data was not extractable for solely nocturnal assessment. This could mean that there are more studies available regarding nocturnal surveillance than what is included in this review, but that the outcomes are not possible to assess in a purely nocturnal setting. The time-to-response for adverse events was also not assessed in this review. This measure may significantly affect health- and welfare-related outcomes by e.g. preventing or reducing the severity of injury through faster responses. Searches were also restricted to Nordic languages in addition to English and French. Searches in additional languages, particularly in the grey literature, may have increased the number of identified studies.

## Conclusions

In summary, there is little evidence to show that nocturnal digital monitoring methods are superior to standard care in several relevant outcomes for stakeholders and end-users. If the main motivation to use digital monitoring is to reduce costs of care services, then the monitoring system would need to be as least as effective as standard care, but evidence of this is lacking due to a small number of high-quality assessment and evaluation studies.

## Supplementary Information

**Additional file 1.**

## Data Availability

The datasets used and/or analysed during the current study are available from the corresponding author on reasonable request.

## Abbreviations

NICE: National Institute for Health and Care Excellence, England
RCT: Randomized Controlled Trial
