# Supplementary material for: Nocturnal digital surveillance in aged populations and its effects on health, welfare and social care provision: a systematic review
Source: BMC Health Serv Res. 2021 Jun 30;21:622. doi: 10.1186/s12913-021-06624-9 (PMC8241531; doi:10.1186/s12913-021-06624-9)
Supplement: Supplementary file 1 — Additional file 1. [file 12913_2021_6624_MOESM1_ESM.docx]

**Supplement 1. List of established grey literature sources searched (September 2020).**

**International registers and databases of grey literature**

OpenGrey ([www.opengrey.eu](https://studentmdh-my.sharepoint.com/personal/matt_richardson_mdh_se/Documents/Systematic%20Reviews/Night%20surveillance/Manuset/www.opengrey.eu))

OAlster (oalster.worldcat.org),

Bielefeld Academic Search Engine – BASE (www.base-search.net),

WHO ICTRP (apps.who.int/trialsearch),

ClinicalTrials.gov ([clinicaltrials.gov/ct2/home](https://studentmdh-my.sharepoint.com/personal/matt_richardson_mdh_se/Documents/Systematic%20Reviews/Night%20surveillance/Manuset/clinicaltrials.gov/ct2/home)),

International Health Technology Assessment Database (<https://www.inahta.org/hta-database>),

DART-Europe ([www.dart-europe.eu/basic-search.php](https://studentmdh-my.sharepoint.com/personal/matt_richardson_mdh_se/Documents/Systematic%20Reviews/Night%20surveillance/Manuset/www.dart-europe.eu/basic-search.php)),

Dissertations and Theses A&I (ProQuest)

Google Scholar (in the respective languages listed in the review methodology)

**National registers and databases of grey literature in the Nordic countries**

Organisations responsible for elderly care, digitalization in health and welfare, and related issues:

The Swedish Board of Health and Welfare: <https://www.socialstyrelsen.se/>

The Swedish Agency for Health and Care Services Analysis: <https://www.vardanalys.se/>

The Swedish Health and Social Care Inspectorate: <https://www.ivo.se/>

The Swedish Post and Telecom Authority: <https://www.pts.se/>

The Swedish Data Protection Authority: <https://www.datainspektionen.se/>

The Swedish Civil Contingencies Agency: <https://www.msb.se/>

Vinnova, a Swedish Agency for Enterprise and Innovation: <https://www.vinnova.se/>

The Swedish Agency for Participation: <https://www.mfd.se/>

The Swedish Agency for e-Health: <https://www.ehalsomyndigheten.se/>

The Swedish Public Health Agency: <https://www.folkhalsomyndigheten.se/>

The Swedish Gender Equality Agency: <https://www.jamstalldhetsmyndigheten.se/>

The Swedish Agency for Digital Government: <https://www.digg.se/>

The Swedish Institute for Standards: <https://www.sis.se/>

The Swedish National Agency for Public Procurement: <https://www.upphandlingsmyndigheten.se/>

The Icelandic Directorate of Health: <https://www.landlaeknir.is/english/>

The Danish National Board of Social Services: <https://socialstyrelsen.dk/>

The Danish Agency for Digitisation: <https://digst.dk/>

The Norwegian Ministry of Health and Care Services - <https://www.regjeringen.no/no/dep/hod/id421/>

The Norwegian Directorate of Health: <https://www.helsedirektoratet.no/>

The Norwegian Directorate of e-Health: <https://ehelse.no/>

The Norwegian Institute of Public Health: <https://www.fhi.no/>

The Finnish Ministry of Social Affairs and Health: <https://stm.fi/sv/framsida>

The Finnish National Institute for Health and Welfare: <https://thl.fi/en/web/thlfi-en>

Muncipalities’, countries’ and regions’ central organisations:

The Swedish Association of Local Authorities and Regions: <https://skr.se/>

Local Government Denmark: <https://www.kl.dk/>

The Norwegian Association of Local and Regional Authorities: <https://www.ks.no/>

The Finnish Association of Local and Regional Authorities: <https://www.kommunforbundet.fi/>

The Icelandic Association of Local Authorities - <https://old.samband.is/english/>

Publication sites owned by universities and colleges:

SwePub (Sweden): <http://swepub.kb.se/>

CRIStin (Norway): <http://www.cristin.no>

Juuli (Finland): <http://www.juuli.fi/?&lng=sv>

Forskningsdatabasen (Danmark): <http://www.forskningsdatabasen.dk>

Opin visindi (Iceland): <https://opinvisindi.is/>

Other research-based institutes and organisations:

The Nordic Welfare Centre: <https://nordicwelfare.org/>

SINTEF (Norway): <https://www.sintef.no/>

RISE (Sweden): <https://www.ri.se/sv>

The Danish National Network for Welfare Technologies: <https://www.carenet.nu/>

Welfare Tech (Denmark): <https://en.welfaretech.dk/>

Nasijonalt senter for e-helseforskning (Norway): <https://ehealthresearch.no>

**Supplement 2. Data extraction template for publications included in the systematic review.**

# **General information**

Grey literature: (Yes/No)

Title:

Country in which the study was conducted:

# **Characteristics of included studies**

## **Methods**

Aim of study:

Study design:

Details regarding study design (optional):

Start date:

End date:

Study funding sources:

Possible conflicts of interest for study authors:

# **Population(s)**

Population(s) description:

Inclusion criteria:

Exclusion criteria:

Method of recruitment of participants:

Total number of participants:

# **Intervention(s)**

Intervention (of relevance) description:

Other interventions (not of relevance):

# **Comparison(s)**

Comparison(s) description:

# **Outcome(s)**

Outcome(s) (of relevance) description:

Other outcome(s) (not of relevance):

# **Data**

All relevant extractable outcome data:

Description of relevant outcome data could not be extracted:

Description of other non-relevant outcome data:
